# Influencing factors of pregnancy loss and survival probability of clinical pregnancies conceived through assisted reproductive technology

**DOI:** 10.1186/s12958-018-0390-6

**Published:** 2018-08-07

**Authors:** Lingmin Hu, Jiangbo Du, Hong Lv, Jing Zhao, Mengxi Chen, Yifeng Wang, Fang Wu, Feng Liu, Xiaojiao Chen, Junqiang Zhang, Hongxia Ma, Guangfu Jin, Hongbing Shen, Li Chen, Xiufeng Ling, Zhibin Hu

**Affiliations:** 1 Department of Reproduction, the Affiliated Changzhou Maternity and Child Health Care, Hospital of Nanjing Medical University, Changzhou, 213003 Jiangsu China; 2 Department of Epidemiology, School of Public Health, Nanjing Medical University, Nanjing, 211166 China; 3 State Key Laboratory of Reproductive Medicine, Nanjing Medical University, Nanjing, 211166 China; 4 Department of Reproduction, the Affiliated Nanjing Maternity and Child Health, Hospital of Nanjing Medical University, Nanjing, 210004 China

**Keywords:** Abortion, Spontaneous, Reproductive techniques, Assisted, Kaplan-Meier estimate

## Abstract

**Background:**

Pregnancies following assisted reproductive technology (ART) may have elevated potential risk of pregnancy loss (PL) when compared to natural conception. However, rare studies comprehensively analyzed the IVF/ICSI cycle-dependent factors for loss of clinical pregnancy. Therefore, we aimed to determine the ART subgroup-specific risks of PL throughout pregnancy and explore different risk factors for early miscarriage and late miscarriage among pregnancies conceived through ART.

**Methods:**

A retrospective cohort study was launched in two infertility treatment centers in Nanjing and Changzhou including 5485 IVF/ICSI embryo transfer cycles with known outcomes after clinical pregnancy by the end of 2015. Cox proportional hazards regression analysis was performed to estimate the hazard ratios and their 95% confidence intervals. The associations between survival time during pregnancy and demographics and clinical characteristics of clinical pregnancies were estimated using the Kaplan-Meier method and the Log-rank test.

**Results:**

The overall PL rate in current ART population was 12.5%. Among the 685 pregnancy loss cycles, a total of 460 ended as early miscarriage, 191 as late miscarriage. We found couples in ART pregnancies demonstrated a significantly increased risk of PL as maternal age (HR = 1.31, *P*_trend_ < 0.001) grows. Pregnancies received controlled ovarian hyperstimulation (COH) protocol like GnRH antagonist protocol (HR = 3.49, *P* < 0.001) and minimal stimulation protocol (HR = 1.83, *P* < 0.001) had higher risk of PL than GnRH-a long protocol. Notably, in contrast to fresh cycle, women who received frozen cycle embryo had a significant increased risk of early miscarriage (*P* < 0.001), while frozen cycle was linked with lower risk of late miscarriage (*P* = 0.045). In addition, four factors (maternal age, COH protocol, cycle type and serum hCG level 14 days after transfer) had independent impact on miscarriage mainly before 12 weeks of gestational age.

**Conclusions:**

With these findings in this study, clinicians may make it better to evaluate a patient’s risk of PL based on the maternal age at the time of treatment, COH protocol, cycle type and serum hCG level 14 days after transfer and the gestational week of the fetus, and we hope that it contributes to future study on its etiology and guide the clinical prevention and treatment.

**Electronic supplementary material:**

The online version of this article (10.1186/s12958-018-0390-6) contains supplementary material, which is available to authorized users.

## Background

Nearly one in six couples will encounter with fertility problems, defined as failure to achieve a clinical pregnancy for 12-month delay [1]. Steadily increasing numbers of couples are turning to assisted reproductive technology (ART) for help, such as in vitro fertilization (IVF) or intracytoplasmic sperm injection (ICSI), conceiving and ultimately giving birth to a healthy live baby of their own. Although the clinical pregnancy rate was gradually improved over the past decade, up to 46.9% reported to Centers for Disease Control (CDC) in the United States by the end of 2012, but the rate of live birth was still low, only 38.1% [2]. Therefore, pregnancy loss (PL), including the loss of a desired pregnancy by miscarriage, stillbirth or termination for genetic indications [3], significantly threatens the rate of live-birth delivery.

The PL rate in natural conception was reported 10%-16% [4–6], while ART pregnancies might have increased potential probability of loss [2, 5, 7]. Data on 148,494 ART pregnancies in United States conceived from 1999 through 2002 had indicated that the PL rate in ART was up to 29% [7]. The potential risk of pregnancy loss in natural conceived conception was mainly determined by elder maternal age (≥35 years) [8], overweight or obese [9], history of abortion [4], microbial infections [10] and elevated reproductive hormones [11]. As for ART population, the elevated potential risk of loss had also been related to some potentially factors specific to women with infertility, such as fresh or frozen cycle type, uterine factor [12], polycystic ovary syndrome (PCOS) status [13]. However, additional unknown barriers may affect the efficiency of ART treatment and require further research. Therefore, it is essential to determine IVF/ICSI cycle-dependent factors for miscarriage and stillbirth, which has important clinical implications for the ART success rates elevation and may help understand possible mechanisms of abortion, thereby improving assisted reproduction technology and strategy.

Miscarriage or spontaneous abortion (SA) is defined as the spontaneous loss of a pregnancy during the first 24 completed weeks of gestational age and it accounted for 80% of fetal losses. Early miscarriage refers to pregnancy loss before 12 weeks of gestational age, while late miscarriage occurs between 12 weeks and 24 weeks [10]. Previous studies revealed that causative factors differed in early miscarriages and late miscarriages, and most early miscarriages resulted from aneuploidy that was greatly influenced by total parental age [14], while late miscarriages were attributed to antiphospholipid syndrome, congenital uterine anomalies, cervical weakness, infection and placental insufficiency [15]. Although many studies have assessed risk factors for early miscarriage in ART pregnancies [12, 16, 17], rare large scale studies have explored the risk factor differences between early SA and late SA among pregnancies conceived through ART. This led us to investigate different risk factors between them, which may be applied to counsel ART pregnant women about their risk time period of SA and help clarify the pathogenesis of miscarriage in order to guide the clinical prevention and ART effective treatment. In addition, previous studies utilizing cross-sectional data only discussed the relation of risk factors and miscarriages, but these studies did not explain the effect of time on ART outcomes.

Therefore, the objectives of current study were to examine PL rates and IVF/ICSI cycle-dependent factors influencing live birth probability as pregnancy progresses on specific subgroups by survival analysis method and investigate the different risks of early miscarriage and late miscarriage after following IVF/ICSI treatment.

## Methods

By the end of 2015, a total of 5856 embryo transfer (ET) cycles carried out and resulted in clinical pregnancies in Reproductive Medicine Center of the Affiliated Nanjing Maternity and Child Health Hospital of Nanjing Medical University (Nanjing) and Reproductive Medicine Center of the Affiliated Changzhou Maternity and Child Health Care Hospital of Nanjing Medical University (Changzhou). We restricted the analysis to cycles with records about final pregnancy outcome (pregnancy loss or live-birth). We excluded donor/preimplantation genetic diagnosis (PGD)/preimplantation genetic screening (PGS) cycles (*N* = 2) and natural ET cycles (*N* = 18). In addition, cycles were excluded if patients were diagnosed with abnormal karyotypes (*N* = 23) or uterine anomalies like fibroids, persistent müllerian duct syndrome or asherman syndrome (*N* = 87). 241 cycles were excluded because of missing data of gestational weeks. After all exclusions, 4165 cycles in Nanjing and 1320 in Changzhou were available for analysis.

Detailed information on maternal and paternal characteristics, ART treatment procedures were collected from the electronic medical records of the two reproductive centers. The pregnancy outcomes were obtained from the follow-up database. Pregnancy was defined as positive serum human chorionic gonadotropin (hCG) level on day 14 after oocyte retrieval, and clinical pregnancy referred to visualization of a gestational sac on ultrasound 3-4weeks after positive hCG test. The gestational week was equal to survival months during pregnancy.

The risk factors for pregnancy loss investigated in this study were maternal age, maternal BMI (body mass index, kg/m^2^), paternal BMI, infertility type, controlled ovarian hyperstimulation (COH) protocol, the total gonadotropin (Gn) dose, fertilization methods, cycle type, no. of embryos transferred, cleavage-stage embryo or blastocyst, serum hCG level 14 days after transfer (IU/L).

Maternal age, maternal BMI, paternal BMI and no. of embryos transferred were categorized for the clarity of data analysis. Maternal age was divided into four subgroups (<30 years, 30-35 years, 36-40 years, >40years). Maternal BMI and paternal BMI subgroups were: <18.5 kg/m^2^, 18.5-24.9 kg/m^2^, 25-28 kg/m^2^, >28 kg/m^2^. Primary infertility was defined as the inability to achieve a clinical pregnancy after 12 months of unprotected and regular sexual intercourse when a woman has never conceived, while secondary infertility was the incapability to conceive in a couple who have had at least one successful clinical pregnancy previously. Three subgroups of no. of embryos transferred were: Group 1(1 embryo transferred), Group 2 (2 embryos transferred) and Group 3 (3 embryos transferred). Depending on the usage of a gonadotropin-releasing hormone agonist (GnRH-a) versus antagonist analogue, GnRH analogue ART protocols are classified as GnRH-a or GnRH antagonist protocols. Among the various GnRH-a protocols, including long, short and rolonged protocol, GnRH-a long protocol is the most conventional protocol. Another protocol utilizes the usage of clomiphene citrate (CC) in combination with Gn, which is termed minimal stimulation protocol. Thus, COH protocols were divided into six categories in this study, including GnRH-a long protocol, GnRH-a short protocol, GnRH antagonist protocol, minimal stimulation protocol, GnRH-a rolonged protocol and other protocol. Besides among them, GnRH antagonist protocol was not utilized in Changzhou, and they only transferred cleavage-stage embryos. 2 days and 3 days embryo before transfer belong to cleavage-stage embryo, while 5 days and 6 days embryo before transfer belong to blastocyst.

### Statistical methods

Demographics and clinical characteristics of pregnancies conceived through ART were calculated by chi-square test. The distributions of continuous variables were evaluated by using Wilcox-test. The associations between survival time during pregnancy and demographics and clinical characteristics of clinical pregnancies were estimated using the Kaplan-Meier method and the Log-rank test. Cox proportional hazards regression analysis was performed to estimate the hazard ratio (HR) and 95% confidence interval (CI). *P* values were given for two-sided tests and statistical significance was defined by *P* < 0.05. Superscript asterisk (*) was added after *P* values with significnce in Tables. Pregnancy survival curves were constructed by the Kaplan-Meier method [18]. Pregnancies were right-censored at completion of the 26^th^ week, because above 98% pregnancy loss were occurred before the 26^th^ week while all of live births occurred between 26 and 42 weeks’ gestation. All the statistical analyses were carried out by R software (Version 3.0.2, 2013-09-25; R Foundation for Statistical Computing, http://www.cran.r-project.org/).

## Results

### Demographics and clinical characteristics of clinical pregnancies

Table 1 summarized demographics and clinical characteristics of clinical pregnancies conceived through ART and compared the differences of these demographics and clinical characteristics between Nanjing and Changzhou. We found that compared with clinical pregnancies in Nanjing, those in Changzhou had significantly higher maternal age (30.42±4.03 vs. 29.89±4.03, *P* < 0.001), and serum hCG level 14 days after transfer (861.75±568.97 vs. 619.49±470.08, *P* < 0.001), lower the total Gn dose in cycle (1728.88±788.45 vs. 1833.67±613.50, *P* < 0.001). The mean of total Gn dose in cycle and serum HCG level 14 days after transfer were 1808.01 IU and 695.30 IU/L respectively, thereby setting as the cutoff values. Besides, there were statistically highly significant distribution differences of six COH protocols, fertilization methods, cycle type, no. of embryos transferred and cleavage-stage embryo or blastocyst among pregnancies between Nanjing and Changzhou (all *P* < 0.001), whereas no difference of infertility type , maternal BMI and paternal BMI were found.

**Table 1:** Demographics and clinical characteristics of clinical pregnancies conceived through ART

Variables	Total	Nanjing	Changzhou	*P*
Total number	5485	4165	1320	
Maternal age	30.01 ± 4.03	29.89 ± 4.03	30.42 ± 4.03	*<0.001* ^*^
Maternal BMI	22.08 ± 3.03	22.04 ± 2.99	22.21 ± 3.16	0.227
Paternal BMI	24.23 ± 3.09	24.18 ± 2.98	24.37 ± 3.39	0.131
Infertility type				0.593
Primary	2781(53.0%)	2165(52.8%)	616(53.7%)	
Secondary	2466(47.0%)	1935(47.2%)	531(46.3%)	
COH protocol				*<0.001* ^*^
GnRH-a long protocol	3406(63.1%)	2311(56.7%)	1095(83.0%)	
GnRH-a short protocol	1573(29.2%)	1448(35.5%)	125(9.5%)	
GnRH antagonist protocol	95(1.8%)	95(2.3%)	0(0.0%)	
Minimal stimulation protocol	186(3.4%)	111(2.7%)	75(5.7%)	
GnRH-a rolonged protocol	123(2.3%)	108(2.6%)	15(1.1%)	
Other protocol	13(0.2%)	4(0.1%)	9(0.7%)	
Total Gn dose	1808.01 ± 662.11	1833.67 ± 613.50	1728.88 ± 788.45	*<0.001* ^*^
Groups of total Gn dose				*<0.001* ^*^
< 1808.01	3181(59.0%)	2344(57.6%)	837(63.4%)	
≥ 1808.01	2208(41.0%)	1725(42.4%)	483(36.6%)	
Fertilization methods				*<0.001* ^*^
IVF	4543(83.9%)	3641(88.8%)	902(68.3%)	
ICSI	875(16.1%)	457(11.2%)	418(31.7%)	
Cycle type				*<0.001* ^*^
Fresh	2675(51.9%)	2202(57.5%)	473(35.8%)	
Frozen	2477(48.1%)	1630(42.5%)	847(64.2%)	
No. of embryos transferred				*<0.001* ^*^
1	474(8.7%)	442(10.7%)	32(2.4%)	
2	4324(79.6%)	3088(75.0%)	1236(93.6%)	
3	655(11.5%)	585(14.2%)	52(3.9%)	
Cleavage-stage embryo or blastocyst				
Cleavage-stage embryo	3860(73.3%)	2540(64.4%)	1320(100.0%)	
Blastocyst	1405(26.7%)	1405(35.6%)	0(0.0%)	
Serum hCG levels 14 days after transfer	695.30 ± 515.44	619.49 ± 470.08	861.75 ± 568.97	*<0.001* ^*^
Groups of serum hCG levels 14 days after transfer				*<0.001* ^*^
< 695.30	2205(52.2%)	1598(55.5%)	607(46.3%)	
≥ 695.30	1988(47.8%)	1283(44.5%)	705(53.7%)	

### Factors influencing live birth probability throughout gestational weeks

As shown in Table 2, the overall rate of pregnancy loss in IVF/ICSI clinical pregnancies was 12.5% (685/5485). Maternal age, maternal BMI, COH protocol, cycle type, no. of embryos transferred and cleavage-stage embryo or blastocyst were significantly associated with the survival time during pregnancy by log-rank test after adjusted by maternal age (all log-rank *P* < 0.05, data not shown).

**Table 2:** Cox analysis of risk of pregnancy loss throughout pregnancy in ART clinical pregnancies

Variables	No. of PL/total (685/ 5485)	PL rate	HR (95% CI)^a^	*P* ^*a*^
Maternal age				
< 30	292/2556	11.4%	1.00[Reference]	
30–35	283/2361	12.0%	1.06(0.90–1.25)	0.508
36–40	84/472	17.8%	1.63(1.28–2.08)	*<0.001* ^*^
> 40	20/53	37.7%	4.14(2.63–6.52)	*<0.001* ^*^
P_trend_			1.31(1.17–1.45)	*<0.001* ^*^
Maternal BMI				
< 18.5	50/509	9.8%	0.85(0.63–1.13)	0.260
18.5–25	487/4009	12.1%	1.00[Reference]	
25–28	97/641	15.1%	1.24(1.00–1.55)	0.051
≥ 28	42/235	17.9%	1.52(1.11–2.10)	*0.010* ^*^
Paternal BMI				
< 18.5	9/103	8.7%	0.67(0.33–1.35)	0.264
18.5–25	405/3309	12.2%	1.00[Reference]	
25–28	189/1344	14.1%	1.15(0.96–1.37)	0.120
≥ 28	69/603	12.3%	0.95(0.74–1.23)	0.691
Infertility type				
Primary	337/2781	12.1%	1.00[Reference]	
Secondary	319/2466	12.9%	0.99(0.85–1.16)	0.908
COH protocol^d^				
GnRH-a long protocol	391/3406	11.5%	1.00[Reference]	
GnRH-a short protocol	186/1573	11.8%	0.93(0.77–1.11)	0.373
GnRH antagonist protocol	35/95	36.8%	3.49(2.46–4.94)	*<0.001* ^*^
Minimal stimulation protocol	44/186	23.7%	1.83(1.31–2.54)	*<0.001* ^*^
GnRH-a rolonged protocol	12/123	9.8%	0.84(0.48–1.50)	0.689
Other protocol	5/13	38.5%	3.70(1.53–8.95)	*0.004* ^*^
The total Gn dose^b^				
< 1808.01	373/3181	11.7%	1.00[Reference]	
≥ 1808.01	301/2208	13.6%	1.12(0.96–1.30)	0.161
Fertilization methods				
IVF	579/4543	12.7%	1.00[Reference]	
ICSI	98/875	11.2%	0.87(0.70–1.08)	0.205
Cycle type				
Fresh	282/2675	10.5%	1.00[Reference]	
Frozen	337/2477	13.6%	1.30(1.11–1.53)	*0.001* ^*^
No. of embryos transferred				
1	82/474	17.3%	1.00[Reference]	
2	516/4324	11.9%	0.71(0.56–0.90)	*0.005* ^*^
3	84/637	13.2%	0.67(0.49–0.91)	*0.010* ^*^
P_trend_			0.81(0.69–0.96)	*0.012* ^*^
Cleavage-stage embryo or blastocyst^d^				
Cleavage-stage embryo	466/3860	12.1%	1.00[Reference]	
Blastocyst	198/1405	14.1%	1.10(1.01–1.19)	*0.030* ^*^
Serum hCG levels 14 days after transfer^c^				
< 695.30	334/2205	15.1%	1.00[Reference]	
≥ 695.30	115/1988	5.8%	0.36(0.29–0.45)	*<0.001* ^*^

^a^Adjusted for maternal age

^b^The cutoff value was the mean of total Gn dose in cycle

^c^The cutoff value was the mean of serum hCG levels 14 days after transfer

^d^GnRH antagonist protocol was not utilized in Changzhou, and they only transferred cleavage-stage embryos

We found couples in ART pregnancies demonstrated a significantly increased risk of PL as maternal age (HR=1.31, 95% CI=1.17-1.45, *P*_trend_<0.001) grows when age was divided into four subgroups. Moreover, compared with women <30 years old, those aged 36-40 years (HR=1.63, 95% CI=1.28-2.08, *P* < 0.001) and older than 40 years (HR=4.14, 95% CI=2.63-6.52, *P* < 0.001) had significantly shorter survival pregnancy weeks. Besides, obese women (BMI≥28, HR=1.52, 95% CI=1.11-2.10, *P* = 0.010) tended to have higher risk of PL compared with the normal BMI group (BMI=18.5-25), yet there was significant heterogeneity for maternal BMI (BMI≥28) in two reproductive medicine centers (Nanjing and Changzhou) based on heterogeneity test (*P* for heterogeneity test was 0.02) (Additional file 1: Table S1). What’s more, pregnancies received COH protocol like GnRH antagonist protocol (HR=3.49, 95% CI=2.46-4.94, *P* < 0.001) and minimal stimulation protocol (HR=1.83, 95% CI=1.31-2.54, *P* < 0.001) had higher risk of PL than GnRH-a long protocol.

Cycle type was significantly associated with the survival time during pregnancy by Cox analysis adjusted for maternal age as well (Log-rank *P* < 0.001). Notable, the risk of loss increased after frozen cycles (HR=1.30, 95% CI=1.11-1.53, *P* = 0.001). However, heterogeneity was also significant for cycle type (*P* = 0.011) between two reproductive medicine centers (Nanjing and Changzhou). After two and three embryos transfer, couples demonstrated 29% (HR=0.71, 95% CI=056-0.90, *P* = 0.005) and 33% (HR=0.67, 95% CI=0.49-0.91, *P* = 0.010) decreased risk of PL than those after one embryos transfer. And, a significant locus-dosage effect was detected between three groups and risk of PL (adjusted HR=0.81, 95% CI=0.69-0.96, *P*_trend_=0.012). In addition, pregnancies after blastocyst transfer had higher risk than those after cleavage-stage embryo transfer after accounting for maternal age (HR=1.10, 95% CI=1.01-1.19, *P* = 0.030). However, after stratification analysis, we found only cleavage-stage embryos were transferred in Changzhou and the difference was no more significant. Moreover, on day 14 after transfer, women with hCG≥695.30 IU/L had an HR of 0.36 (95% CI=0.29-0.45, *P* < 0.001) compared to those with hCG lower than 695.30 IU/L.

**Fig. 1:**
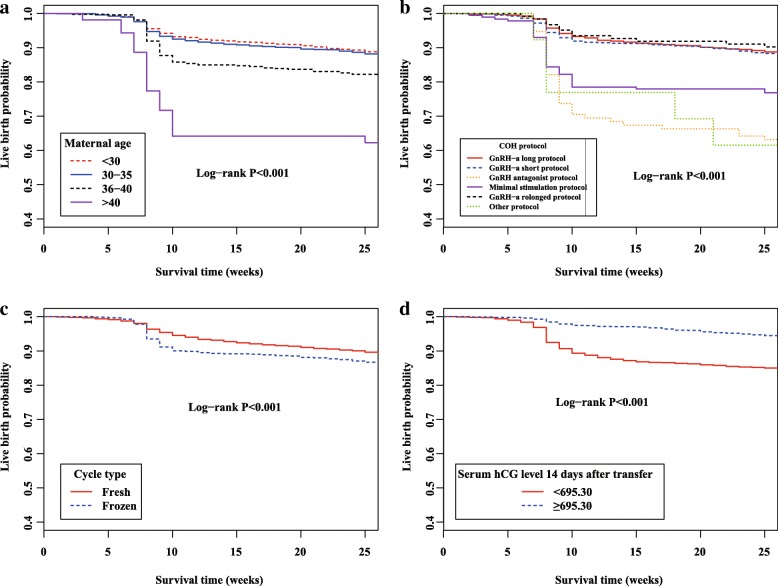
Kaplan-Meier plots of live birth by maternal age (**a**), COH protocol (**b**), cycle type (**c**) and serum hCG levels 14 days after transfer (**d**)

To get insight into the independent effects of characteristics and clinical features of pregnancies on survival time during ART pregnancy, we performed stepwise backward Cox proportional hazard analysis. As shown in Table 3, four independent factors were determined, including maternal age (compared with maternal age<30, *P* = 0.027 for maternal age between 36 and 40 years, *P* < 0.001 for maternal age>40 years), COH protocol (compared with GnRH-a long protocol, *P* value<0.001 for both GnRH antagonist protocol and mini-stimulation protocol), cycle type (*P* = 0.030) and serum hCG level 14 days after transfer (*P* < 0.001). Kaplan-Meier plots of live birth by maternal age, COH protocol, cycle type and serum hCG level 14 days after transfer were shown in Fig. 1.

**Table 3:** Stepwise Cox regression analysis on live birth probability of ART clinical pregnancies

Variables	β^a^	SE^b^	*HR*	95% CI	*P*
Maternal age (reference: <30)					
30–35	0.0733	0.1102	1.08	0.87–1.34	0.506
36–40	0.3758	0.1697	1.46	1.04–2.03	*0.027* ^*^
> 40	1.1000	0.3222	3.00	1.60–5.65	*<0.001* ^*^
COH protocol (reference: GnRH-a long protocol)					
GnRH-a short protocol	0.0326	0.1191	1.03	0.82–1.30	0.784
GnRH antagonist protocol	0.8914	0.2698	2.44	1.44–4.14	*<0.001* ^*^
Minimal stimulation protocol	0.7869	0.2152	2.20	1.44–3.35	*<0.001* ^*^
GnRH-a rolonged protocol	−0.1192	0.4160	0.89	0.39–2.01	0.774
Other protocol	1.3692	0.5070	3.93	1.46–10.62	*0.007* ^*^
Cycle Type (reference: Fresh)					
Frozen	0.2296	0.1057	1.26	1.02–1.55	*0.030* ^*^
Serum hCG levels 14 days after transfer (reference: < 695.30)					
≥ 695.30	−1.0848	0.1180	0.34	0.27–0.43	*<0.001* ^*^

*HR* hazard ratio, *CI* confidence interval

^a^β is the estimated parameter of the regression model

^b^SE is the standard error of the regression model

### Risk factors for early miscarriage and late miscarriage

In Table 4, among the 685 pregnancy loss cycles, a total of 460 ended as early SA, 191 as late SA. The rate of early miscarriage in ART clinical pregnancies was 8.4% (460/5485) while the rate of late miscarriage in ART clinical pregnancies after 12 weeks of gestational age was 3.8% (191/5025). Of those clinical pregnancies, women aged 36-40 years (HR=2.14, 95% CI=1.62-2.83, *P* < 0.001) and over 40 years (HR=6.04, 95% CI=3.76-9.69, *P* < 0.001) were more likely to encounter early pregnancy loss in contrast to those younger than 30 years old. Besides, treated by GnRH antagonist and minimal stimulation COH protocol (both *P* < 0.001), blastocyst transferred (*P* = 0.006), and frozen embryo transferred (*P* < 0.001) were more likely to encounter early pregnancy loss. What’s more, there was a significant decrease of risk of early SA in pregnancies after two embryos transferred compared with those following one embryo transfer (*P* < 0.001). Serum hCG level at higher level on day 14 after transfer was linked with a significant 78% and 32% decreased risk of early SA and late SA (*P* < 0.001). Moreover, maternal BMI≥28 had a positive impact on late PL rather than early PL. Notably, in contrast to fresh cycle, women who received frozen cycle embryo had a significant decrease of risk of late SA (*P* = 0.045), while frozen cycle was linked with higher risk of early PL. After stepwise backward Cox proportional hazard analysis, we found that maternal age, COH protocol, cycle type and hCG level 14 days after transfer had independent effects on live birth probability of ART clinical pregnancies before 12 weeks of gestational age (In Table 5). However, none of factors for PL investigated in this study had independent effects on late miscarriage (data not shown).

**Table 4:** Comparison of early miscarriage and late miscarriage in ART clinical pregnancies

Variables	Early miscarriage				Late miscarriage			
	N^c^ (%)	n (%)	HR (95%CI)^a^	*P* ^a^	N^d^ (%)	n (%)	HR (95%CI)^a^	*P* ^a^
Maternal age								
< 30	2556(47.0)	180(39.5)	1.00		2376(47.7)	94(49.7)	1.00	
30–35	2361(43.4)	188(41.2)	1.14(0.93–1.40)	0.215	2173(43.6)	80(42.3)	0.93(0.69–1.25)	0.630
36–40	472(8.7)	69(15.1)	2.14(1.62–2.83)	*<0.001* ^*^	403(8.1)	15(7.9)	0.94(0.54–1.62)	0.823
> 40	53(1.0)	19(4.2)	6.04(3.76–9.69)	*<0.001* ^*^	34(0.7)	0(0.0)	0	0.992
Maternal BMI								
< 18.5	509(9.4)	32(7.0)	0.82(0.57–1.18)	0.282	477(9.7)	15(8.1)	0.82(0.52–1.51)	0.647
18.5–25	4009(74.3)	335(73.3)	1.00		3674(74.4)	130(70.3)	1.00	
25–28	641(11.9)	65(14.2)	1.19(0.91–1.55)	0.205	576(11.7)	24(13.0)	1.20(0.78–1.86)	0.407
≥28	235(4.4)	25(5.5)	1.35(0.90–2.03)	0.145	210(4.3)	16(8.6)	2.09(1.22–3.57)	*0.007* ^*^
Paternal BMI								
< 18.5	103(1.9)	8(1.8)	1.07(0.53–2.16)	0.859	95(1.9)	1(0.5)	0.00(0.00 − +∞)	0.992
18.5–25	3309(61.7)	266(58.6)	1.00		3043(62.0)	120(64.9)	1.00	
25–28	1344(25.1)	129(28.4)	1.17(0.95–1.45)	0.137	1215(24.8)	50(27.0)	1.06(0.76–1.48)	0.728
≥ 28	603(11.3)	51(11.2)	1.08(0.80–1.46)	0.616	552(11.3)	14(7.6)	0.64(0.37–1.12)	0.118
Infertility type								
Primary	2781(53.0)	227(51.1)	1.00		2554(53.2)	92(51.7)	1.00	
Secondary	2466(47.0)	217(48.9)	0.94(0.78–1.14)	0.544	2249(46.8)	86(48.3)	1.10(0.81–1.49)	0.538
COH protocol^b^								
GnRH-a long protocol	3406(63.1)	243(53.4)	1.00		3163(64.0)	125(67.9)	1.00	
GnRH-a short protocol	1573(29.2)	132(29.0)	1.00(0.80–1.26)	0.974	1441(29.2)	48(26.1)	0.85(0.60–1.21)	0.370
GnRH antagonist protocol	95(1.8)	29(6.4)	4.11(2.78–6.08)	*<0.001* ^*^	66(1.3)	5(2.7)	1.95(0.80–4.78)	0.144
Minimal stimulation protocol	186(3.4)	40(8.8)	2.39(1.67–3.43)	*<0.001* ^*^	146(3.0)	1(0.5)	0.18(0.02–1.29)	0.088
GnRH-a rolonged protocol	123(2.3)	8(1.8)	0.90(0.44–1.82)	0.765	115(2.3)	3(1.6)	0.67(0.21–2.11)	0.492
Other protocol	13(0.2)	3(0.7)	3.27(1.04–10.23)	*0.042* ^*^	10(0.2)	2(1.1)	5.52(1.36–22.40)	*0.017* ^*^
The total Gn dose								
< 1808.01	3181(59.0)	252(55.4)	1.00		2929(59.4)	104(56.2)	1.00	
≥ 1808.01	2208(41.0)	203(44.6)	1.08(0.89–1.30)	0.440	2005(40.6)	81(43.8)	1.15(0.86–1.54)	0.355
Fertilization methods								
IVF	4543(83.9)	386(84.6)	1.00		4157(83.8)	162(86.6)	1.00	
ICSI	875(16.1)	70(15.4)	0.93(0.72–1.20)	0.562	805(16.2)	25(13.4)	0.81(0.53–1.23)	0.320
Cycle type								
Fresh	2675(51.9)	161(39.1)	1.00		2514(53.0)	106(60.6)	1.00	
Frozen	2477(48.1)	251(60.9)	1.67(1.37–2.04)	*<0.001* ^*^	2226(47.0)	69(39.4)	0.73(0.54–0.99)	*0.045* ^*^
No. of embryos transferred								
1	474(8.7)	64(14.0)	1.00		410(8.2)	16(8.4)	1.00	
2	4324(79.6)	330(72.1)	0.61(0.47–0.80)	*<0.001* ^*^	3994(80.2)	157(82.6)	0.99(0.59–1.65)	0.961
3	637(11.7)	64(14.0)	0.63(0.44–0.89)	*0.010* ^*^	573(11.5)	17(8.9)	0.75(0.38–1.51)	0.425
Cleavage-stage embryo or blastocyst^b^								
Cleavage-stage embryo	3860(73.3)	305(68.2)	1.00		3555(73.8)	135(73.8)	1.00	
Blastocyst	1405(26.7)	142(31.8)	1.15(1.04–1.27)	*0.006* ^*^	1263(26.2)	48(26.2)	1.00(0.85–1.18)	0.994
Serum hCG levels 14 days after transfer								
< 695.30	2187(52.2)	248(82.7)	1.00		1939(49.8)	79(59.8)	1.00	
≥ 695.30	2006(47.8)	52(17.3)	0.22(0.16–0.30)	*<0.001* ^*^	1954(50.2)	53(40.2)	0.68(0.48–0.96)	*0.030* ^*^

^a^Adjusted for maternal age

^b^GnRH antagonist protocol was not utilized in Changzhou, and they only transferred cleavage-stage embryos

^c^the number of clinical pregnancies

^d^the number of clinical pregnancies after 12 weeks of gestational age

**Table 5:** Stepwise Cox regression analysis on live birth probability of ART clinical pregnancies before 12 weeks of gestational age

Variables	β^a^	SE^b^	*HR*	95% CI	*P*
Maternal age (reference: <30)					
30–35	0.1196	0.1392	1.13	0.86–1.48	0.390
36–40	0.5253	0.1956	1.69	1.15–2.48	*0.007* ^*^
> 40	1.3087	0.3324	3.70	1.93–7.10	*<0.001* ^*^
COH protocol (reference: GnRH-a long protocol)					
GnRH-a short protocol	0.2526	0.1460	1.29	0.97–1.71	0.084
GnRH antagonist protocol	1.2436	0.2953	3.47	1.94–6.19	*<0.001* ^*^
Minimal stimulation protocol	1.1429	0.2310	3.14	2.00–4.93	*<0.001* ^*^
GnRH-a rolonged protocol	0.0747	0.5109	1.08	0.40–2.93	0.884
Other protocol	1.4356	0.5868	4.20	1.33–13.27	*0.014* ^*^
Cycle Type (reference: Fresh)					
Frozen	0.5059	0.1287	1.66	1.29–2.13	*<0.001* ^*^
Serum hCG levels 14 days after transfer (reference: < 695.30)					
≥ 695.30	−1.6353	0.1687	0.19	0.14–0.27	*<0.001* ^*^

*HR* hazard ratio, *CI* confidence interval, ^*^P<0.05

^a^β is the estimated parameter of the regression model

^b^SE is the standard error of the regression model

## Discussion

This retrospective cohort study on the risk of PL throughout ART pregnancy included 5485 ART clinical pregnancies with data of gestational weeks, 685 of which suffered pregnancy loss. The main findings of this study were that maternal age, COH protocol, cycle type and hCG level 14 days after transfer significantly affected the reproductive pregnancy loss of ART population. Besides, the incidence of PL decreased as pregnancy progresses, early SA therefore occurred more frequently than the late one. To our knowledge, this is the first retrospective cohort study to extensively explore the different risk factors of early miscarriage and late miscarriage after IVF/ICSI.

The negative effect of maternal reproductive age on the risk of PL has been consistently reported and studied in previous studies [19, 20]. Age of the female is the most important risk factor in determining pregnancy success rates both in natural conception and after ART [4]. It is partly because of obvious decline in ovarian germ cells supply, decrease in oocyte quality and ultimately leading to ovarian reproductive failure [21]. In this study, women>40 years old had approximately 37.7% PL rate, and they showed significant decline in survival probability of ART clinical pregnancies progressed to a live birth compared with those <30 years old. Some studies suggested that the decreased fecundity and fertility rate occurred due in part to decreased follicle reserves [22] and increased aneuploidy [23] in the older female. In addition, chromosome abnormality decreases as pregnancy progresses and occurs mostly before 12 weeks of pregnancy [14], which makes the association of older parental age with early SA credible. Now, pre-implantation genetic screening of embryos prior to transfer may reduce early pregnancy wastage resulting from aneuploidy, but safety and risks of the technology need further investigation.

Controlled ovarian hyperstimulation is a fundamental step of IVF/ICSI and over time IVF techniques have developed to satisfy the needs of fertility patients and the improvement of ART success rate. The present results suggest that for the purpose of increasing survival probabilities of ART pregnancies, the GnRH-a long protocol is a better option for IVF/ICSI stimulation compared to GnRH antagonist and minimal stimulation protocol. Using Gn and CC in ART cycles is associated with chromosomal abnormalities in an IVF embryo and subsequent early miscarriage after transfer [24]. Moreover, patients with poor ovarian reserve prefer considering minimal stimulation protocol, and GnRH antagonist protocols are applied to "poor responders" and women at high-risk of developing OHSS, thus oocyte/embryo quality and development was inferior to that of the agonist group. However, data from earlier studies don’t support the finding and indicate that the usage of GnRH antagonist is not associated with reduction of the likelihood of achieving live birth, compared with GnRH-a protocols [25]. Therefore, further investigation is needed to clarify the matter and future developments have to be focused on the optimization of COH protocols.

As for the association of cycle type and the occurrence of PL, we found the rate of loss, especially early miscarriage, increased after frozen IVF/ICSI cycles. We considered that embryo may inevitably be damaged by cryopreservation technology, which may damage the ability of embryo development and thus result in abortion. However, a meta-analysis showed that this difference did not reach statistical significance [26], and even in a multicenter randomized trial, the opposite result showed in infertile women with PCOS [27]. Thus, it is necessary to examine the effect of hormone level. To our surprise, frozen embryos after implantation showed significant lower late SA probability compared to fresh ones in our findings, which was consistent with the two multicenter randomized trials involving infertile women with the PCOS and ovulatory women [27, 28]. However, after stepwise backward Cox proportional hazard analysis, this different was no more significant, which could be due to difference in cryopreservation protocols, freezing day (cleavage-stage or blastocyst), number or quality of frozen embryos transferred.

Serum hCG has been used to be the main endocrine determinant of ongoing pregnancy, and then whether serum hCG level measured on the 14th day after transfer is sufficient in predicting final live birth outcomes needs to be confirmed. It was a significant observation in our findings that the hCG level of live birth was markedly higher than that of PL especially early abortion, which was agreement with another study that estimates the cutoff value of 50 IU/L (75% sensitivity, 81% specificity, *P* < 0.001) to predict ongoing pregnancy [29] as well. However, no hCG cutoff level had a sensitivity or specificity of 100% for pregnancies, making it essential to continue routine monitoring of ART pregnancy outcomes.

In addition to the above, we found BMI, freezing day (cleavage-stage or blastocyst) and no. of embryos transferred affected PL, but the three factors were not significant any more after stepwise COX regression. The incidence of PL progressively ascended as maternal BMI categories increased, which is consistent with a recent study [30]. And the association of female obesity (BMI>28 kg/m^2^) with a significant decreased possibility of ART baby’s survival after 12 weeks of gestational age was another interesting observation. It is suggested that maternal obesity can impair embryo development and the mechanism that Stella insufficiency in oocytes mediates developmental defects in early embryos has been elucidated in a recent study [31]. Besides, it is explained that the higher risk in subjects with high BMI may be due to the action of a hormone named leptin, which is produced predominantly in the adipose tissue [32].

In our finding, more than one embryo transfer was a protective factor of fetal loss in ART pregnancies after accounting for maternal age and the association of two embryos transfer with a significant decreased risk of early loss is an interesting observation. Although the results of a previous study also suggested that live birth and pregnancy rates following single embryo transfer were lower than those following double embryo transfer, so are the chances of multiple pregnancy including twins [33]. Multiple gestations lead to an increased risk of complications in both the fetuses and the mothers, so the optimal and recommended choice is the limit to the number of embryos to transfer [34].

This study was limited by some data availability as there is no routine ART surveillance system. We could not have data of other PL risk factors like microbial infections to resolve existing data gap. Secondly, the data covered in the article derived from two centers in Nanjing and Changzhou, so we would not obtain sufficient and identical characteristics and clinical features of pregnancies for lack data of some variables in either of centers. Lastly, in this retrospective cohort study, we could not investigate the mechanism of miscarriage during pregnancy weeks and the sample size is not enough, so the large-scale prospective cohort study should be carried out to in China.

## Conclusions

In conclusion, the ultimate aim of assisted reproductive medicine practitioners would still be the improvement of IVF/ICSI efficacy in terms of take-home-baby rates. Therefore, with these findings in this study, clinicians may make it better to evaluate an infertile couple’s risk of PL based on the maternal age at the time of treatment, COH protocol, cycle type and serum hCG level 14 days after transfer and the gestational week of the fetus. Additionally, these factors had significant impact on miscarriage mainly before 12 weeks of gestational age. Hopefully, these findings may offer some suggestions of population risks of pregnancy loss for reproductive health epidemiologists and contribute to future study on its etiology.

## Additional file

Additional file 1: **Table S1.** Cox analysis of risk of pregnancy loss throughout pregnancy in ART clinical pregnancies stratified by two fertility centers. (DOC 103 kb)

## Abbreviations

ART: Assisted reproductive technology
BMI: Body mass index
CC: Clomiphene citrate
CDC: Centers for Disease Control
CI: Confidence interval
COH: Controlled ovarian hyperstimulation
ET: Embryo transfer
Gn: Gonadotropin
GnRH-a: Gonadotropin-releasing hormone agonist
hCG: Human chorionic gonadotropin
HR: Hazard ratio
ICSI: Intracytoplasmic sperm injection
IVF: In vitro fertilization
PCOS: Polycystic ovarian syndrome
PGD: Preimplantation genetic diagnosis
PGS: Preimplantation genetic screening
PL: Pregnancy loss
SA: Spontaneous abortion
