# Supplementary material for: Influencing factors of pregnancy loss and survival probability of clinical pregnancies conceived through assisted reproductive technology
Source: Reprod Biol Endocrinol. 2018 Aug 7;16:74. doi: 10.1186/s12958-018-0390-6 (PMC6081896; doi:10.1186/s12958-018-0390-6)
Supplement: Supplementary file 1 — Table S1. Cox analysis of risk of pregnancy loss throughout pregnancy in ART clinical pregnancies stratified by two fertility centers. (DOC 103 kb) [file 12958_2018_390_MOESM1_ESM.doc]

Table 1. Cox analysis of risk of pregnancy loss throughout pregnancy in ART clinical pregnancies stratified by two fertility centers.

| **Variables** | **Nanjing** | | | |  |  | **Changzhou** | |  |  |
| --- | --- | --- | --- | --- | --- | --- | --- | --- | --- | --- |
|  | **No. of**  **PL /total**  **(534/ 4165)** | **PL rate** | **HR (95% CI)*^a^*** | ***P^a^*** |  | **No. of**  **PL /total**  **(151/ 1320)** | **PL rate** | **HR (95% CI)*^a^*** | ***P^a^*** | ***P^b^*** |
| **Maternal age** |  |  |  |  |  |  |  |  |  |  |
| <30 | 237/1991 | 12.1% | 1.00[Reference] |  |  | 55/565 | 9.9% | 1.00[Reference] |  |  |
| 30-35 | 220/1784 | 12.6% | 1.04(0.87-1.25) | 0.652 |  | 63/577 | 11.4% | 1.13(0.79-1.63) | 0.502 | 0.693 |
| 36-40 | 63/352 | 18.2% | 1.57(1.19-2.08) | **0.001** |  | 21/120 | 17.5% | 1.90(1.15-3.14) | **0.013** | 0.521 |
| >40 | 14/38 | 36.8% | 3.78(2.21-6.49) | **<0.001** |  | 6/15 | 40.0% | 5.58(2.40-12.95) | **<0.001** | 0.448 |
| P_trend_ |  |  | 1.27(1.13-1.43) | **<0.001** |  |  |  | 1.46(1.17-1.82) | **0.001** | 0.273 |
| **Maternal BMI** |  |  |  |  |  |  |  |  |  |  |
| <18.5 | 42/395 | 10.6% | 0.92 (0.67-1.26) | 0.597 |  | 8/114 | 7.0% | 0.60(0.29-1.22) | 0.138 | 0.284 |
| 18.5-25 | 369/3048 | 12.1% | 1.00[Reference] |  |  | 118/961 | 12.6% | 1.00[Reference] |  |  |
| 25-28 | 78/472 | 16.5% | 1.36(1.07-1.74) | **0.013** |  | 19/169 | 11.8% | 0.91(0.56-1.48) | 0.782 | 0.149 |
| ≥28 | 36/163 | 22.1% | 1.93(1.37-2.71) | **<0.001** |  | 6/72 | 8.3% | 0.62(0.25-1.51) | 0.260 | **0.020** |
| **Paternal BMI** |  |  |  |  |  |  |  |  |  |  |
| <18.5 | 5/70 | 7.1% | 0.58(0.24-1.40) | 0.227 |  | 4/33 | 12.1% | 0.97(0.31-3.09) | 0.962 | 0.486 |
| 18.5-25 | 325/2562 | 12.7% | 1.00[Reference] |  |  | 80/747 | 10.7% | 1.00[Reference] |  |  |
| 25-28 | 146/991 | 14.7% | 1.16(0.96-1.42) | 0.127 |  | 43/353 | 12.2% | 1.12(0.77-1.64) | 0.543 | 0.873 |
| ≥28 | 45/420 | 10.7% | 0.84(0.61-1.15) | 0.268 |  | 24/183 | 13.1% | 1.35(0.85-2.14) | 0.203 | 0.094 |
| **Infertility type** |  |  |  |  |  |  |  |  |  |  |
| Primary | 268/2165 | 12.4% | 1.00[Reference] |  |  | 69/616 | 11.2% | 1.00[Reference] |  |  |
| Secondary | 253/1935 | 13.1% | 0.99(0.83-1.18) | 0.876 |  | 66/531 | 12.4% | 1.00(0.70-1.43) | 0.989 | 0.935 |
| **COH protocol^e^** |  |  |  |  |  |  |  |  |  |  |
| GnRH-a long protocol | 274/2311 | 11.9% | 1.00[Reference] |  |  | 117/1095 | 10.7% | 1.00[Reference] |  |  |
| GnRH-a short protocol | 171/1448 | 11.8% | 0.90(0.73-1.10) | 0.304 |  | 15/125 | 12.0% | 0.89(0.50-1.59) | 0.686 | 0.967 |
| GnRH antagonist protocol | 35/95 | 36.8% | 3.36(2.35-4.80) | **<0.001** |  | 0/0 | NA | NA | NA | NA |
| Minimal stimulation protocol | 29/111 | 26.1% | 2.03(1.36-3.02) | **<0.001** |  | 15/75 | 20.0% | 1.47(0.81-2.68) | 0.208 | 0.379 |
| GnRH-a rolonged protocol | 11/108 | 10.2% | 0.84(0.46-1.53) | 0.563 |  | 1/15 | 6.7% | 0.68(0.10-4.91) | 0.705 | 0.847 |
| Other protocol | 2/4 | 50.0% | 5.40(1.34-21.71) | **0.017** |  | 3/9 | 33.3% | 3.16(0.99-10.03) | 0.051 | 0.561 |
| **The total Gn dose^c^** |  |  |  |  |  |  |  |  |  |  |
| <1808.01 | 272/2344 | 11.6% | 1.00[Reference] |  |  | 101/837 | 12.1% | 1.00[Reference] |  |  |
| ≥1808.01 | 251/1725 | 14.6% | 1.23(1.03-1.46) | **0.020** |  | 50/483 | 10.4% | 0.74(0.52-1.06) | 0.097 | **0.012** |
| **Fertilization methods** |  |  |  |  |  |  |  |  |  |  |
| IVF | 472/3641 | 13.0% | 1.00[Reference] |  |  | 107/902 | 11.9% | 1.00[Reference] |  |  |
| ICSI | 54/457 | 11.8% | 0.90(0.68-1.19) | 0.449 |  | 44/418 | 10.5% | 0.91(0.63-1.30) | 0.600 | 0.958 |
| **Cycle type** |  |  |  |  |  |  |  |  |  |  |
| Fresh | 225/2202 | 10.2% | 1.00[Reference] |  |  | 57/473 | 12.1% | 1.00[Reference] |  |  |
| Frozen | 243/1630 | 14.9% | 1.49(1.24-1.78) | **<0.001** |  | 94/847 | 11.1% | 0.90(0.65-1.27) | 0.558 | **0.011** |
| **No. of embryos transferred** |  |  |  |  |  |  |  |  |  |  |
| 1 | 75/442 | 17.0% | 1.00[Reference] |  |  | 7/32 | 21.9% | 1.00[Reference] |  |  |
| 2 | 380/3088 | 12.3% | 0.76(0.59-0.97) | **0.030** |  | 136/1236 | 11.0% | 0.48(0.22-1.04) | 0.063 | 0.275 |
| 3 | 76/585 | 13.0% | 0.67(0.49-0.93) | **0.017** |  | 8/52 | 15.4% | 0.58(0.21-1.60) | 0.291 | 0.780 |
| P_trend_ |  |  | 0.82(0.69-0.97) | **0.018** |  |  |  | 0.78(0.42-1.45) | 0.440 | 0.897 |
| **Cleavage-stage embryo or blastocyst^e^** |  |  |  |  |  |  |  |  |  |  |
| cleavage-stage embryo | 315/2540 | 12.4% | 1.00[Reference] |  |  | 151/1320 | 11.4% |  |  |  |
| blastocyst | 198/1405 | 14.1% | 1.08(0.99-1.18) | 0.092 |  |  |  |  |  |  |
| **Serum hCG levels 14 days after transfer^d^** |  |  |  |  |  |  |  |  |  |  |
| <695.30 | 224/1598 | 14.0% | 1.00[Reference] |  |  | 110/607 | 18.1% | 1.00[Reference] |  |  |
| ≥695.30 | 75/1283 | 5.8% | 0.39(0.30-0.51) | **<0.001** |  | 40/705 | 5.7% | 0.29(0.20-0.43) | **<0.001** | 0.208 |

^a^ Adjusted for maternal age.

*^b^* Heterogeneity test for differences between Nanjing and Changzhou.

^c^ The cutoff value was the mean of total Gn dose in cycle.

^d^ The cutoff value was the mean of serum hCG levels 14 days after transfer.

^e^ GnRH antagonist protocol was not utilized in Changzhou, and they only transferred cleavage-stage embryos.
